# Recent advances in bariatric surgery: a narrative review of weight loss procedures

**DOI:** 10.1097/MS9.0000000000001472

**Published:** 2023-11-01

**Authors:** Nicholas Aderinto, Gbolahan Olatunji, Emmanuel Kokori, Peter Olaniyi, Timilehin Isarinade, Ismaila Ajayi Yusuf

**Affiliations:** a Department of Medicine and Surgery, Ladoke Akintola University of Technology, Ogbomoso, Oyo State; b Department of Medicine and Surgery, University of Ilorin, Ilorin; c Obafemi Awolowo University Teaching Hospital Complex, Ile-Ife, Nigeria

**Keywords:** bariatric surgery, obesity, weight loss

## Abstract

Bariatric surgery has emerged as a highly effective option for individuals with obesity, offering significant and sustainable weight loss outcomes. This surgical approach involves various procedures that alter the anatomy of the gastrointestinal tract, leading to reduced food intake and nutrient absorption. Established procedures such as sleeve gastrectomy, gastric bypass, adjustable gastric banding, and biliopancreatic diversion with duodenal switch have proven track records. In contrast, emerging options like intragastric balloons, AspireAssist devices, and endoscopic sleeve gastroplasty show promise but require further investigation. Numerous studies have highlighted the remarkable benefits of bariatric surgery, not only in weight loss but also in the resolution of obesity-related comorbidities and significant improvements in quality of life. However, successful outcomes rely on a multidisciplinary approach, encompassing preoperative evaluation, patient selection, comprehensive postoperative care, nutritional support, and psychological counseling. Regular follow-up and adherence to postsurgical recommendations are crucial for sustained weight loss and positive long-term results. As bariatric surgery continues to evolve, tailored procedures based on individual needs and ongoing research hold the potential for even more refined and effective approaches. Through this ongoing advancement, bariatric surgery is poised to offer improved patient outcomes, transforming lives for those grappling with the challenges of obesity.

## Introduction

Highlights

- Bariatric surgery has proven to be highly effective in providing substantial and sustainable weight loss outcomes for individuals struggling with obesity.
- Bariatric surgery not only aids in weight loss but also resolves obesity-related comorbidities and significantly improves patients’ quality of life. Numerous studies have highlighted the remarkable benefits of improving overall health and well-being.
- Successful outcomes in bariatric surgery depend on a comprehensive multidisciplinary approach. This involves preoperative evaluation, patient selection, comprehensive postoperative care, nutritional support, and psychological counseling. Regular follow-up and adherence to postsurgical recommendations are crucial for sustaining weight loss and achieving positive long-term results.

The prevalence of overweight and obesity has reached alarming proportions globally, posing significant global challenges to global health systems^1^. These conditions are characterized by abnormal or excessive fat accumulation, which can adversely affect an individual’s overall health and quality of life^1^. To classify overweight and obesity, the Body Mass Index (BMI) has emerged as a widely used tool, calculated by dividing an individual’s weight in kilograms by the square of their height in meters (kg/m^2^). In adults, a BMI falling within the range of 18.5–24.9 kg/m^2^ is considered normal, while a BMI equal to or greater than 25 kg/m^2^ is categorized as overweight, and a BMI of 30 kg/m^2^ or higher indicates obesity^2^. For children and adolescents aged 2–18, overweight is defined as a BMI between the 85th and 94th percentiles^3^. At the same time, a BMI represents obesity at or above the 95th percentile for the individual’s sex and age^3^. Over the past four decades, the prevalence of obesity has witnessed a worrisome escalation, affecting both the young and the adult populations alike^4^. Childhood obesity rates have surged from less than 1% to 6–8%, while adult obesity has risen from 3% to 11% among males and 6% to 15% among females^4^. Currently, an estimated 1.5 billion adults worldwide grapple with the burdens of being overweight or obese, and this number is projected to soar to 3 billion by 2030, underscoring the urgent need for effective interventions^2^.

The health consequences of obesity are profound and far-reaching, encompassing an increased risk of cardiovascular diseases (the leading cause of mortality in 2012), type 2 diabetes mellitus, musculoskeletal disorders, and certain cancers^2^. In addition to the immediate health impacts, children with obesity face a heightened risk of long-term adverse outcomes, including premature mortality, disability, respiratory complications, fractures, and early-onset chronic medical conditions like hypertension, cardiovascular disease, insulin resistance, and psychological disorders^2^. The cumulative impact of obesity has spurred considerable concern among healthcare professionals and policymakers, necessitating comprehensive and innovative strategies to curb this growing epidemic^3^.

The escalating prevalence of overweight and obesity has become a pressing global health challenge, demanding effective interventions to address its significant impact on public health. Bariatric surgery has emerged as a promising therapeutic avenue for severely obese individuals, offering substantial weight loss and improvements in associated comorbidities^5^. While previous reviews have explored the efficacy of bariatric surgery in addressing obesity, this study seeks to provide an up-to-date and comprehensive analysis of the latest advancements in this field. By consolidating evidence from recent research, we aim to identify novel approaches and emerging techniques that promise to optimize surgical outcomes and improve long-term patient well-being.

## Methodology

We conducted a comprehensive narrative review to explore recent advances in bariatric surgery as a weight loss procedure. Our primary objectives were to identify and analyze the latest developments in bariatric surgery techniques, evaluate their efficacy, safety, and impact on patient outcomes and quality of life, and propose potential areas of future research to enhance the field.

We conducted an exhaustive literature search to identify relevant studies and publications using databases such as PubMed, MEDLINE, Scopus, Web of Science, and Google Scholar. We used combinations of keywords and Medical Subject Headings (MeSH) terms related to bariatric surgery, obesity, weight loss, surgical techniques, patient outcomes, and quality of life.

For inclusion in the review, articles must meet the following criteria:

1. They should be published in peer-reviewed journals and written in English.
2. The studies should focus on recent advances in bariatric surgery, that is, studies published within the last 10 years.
3. Studies reporting original research, clinical trials, systematic reviews, and meta-analyses.
4. Studies providing data on patient outcomes, safety, or quality of life after bariatric surgery were included.

On the other hand, studies were excluded if they:

1. Were published more than 10 years ago.
2. Are written in languages other than English.
3. Primarily focus on nonsurgical weight loss interventions or non-bariatric surgical procedures.
4. Comprise case reports or letters to the editor without original data.

Two independent reviewers extracted data using a predefined data extraction form. Findings from the selected studies were synthesized and presented descriptively. Our review emphasized identifying common trends, gaps in the literature, and areas of innovation in bariatric surgery.

## Types of bariatric surgery procedures

### Sleeve gastrectomy (SG)

Laparoscopic Sleeve Gastrectomy (LSG) is a prominent bariatric procedure that reshapes the stomach into a narrow, tubular structure using five or six upper abdomen ports^6,7^. It involves vascular attachment division, gastric fundus detachment, posterior gastric artery ligation, and stapling near the pylorus, followed by leak testing and omental suturing for leakage prevention^8,9^. LSG induces weight loss by reducing gastric volume, altering hormonal levels (particularly ghrelin and peptide YY), and changing gastric motility^10,11^. Studies by Durmush *et al*.^12^ reported percentage of excess weight loss (%EWL) of 67.1% at 6 months, 81.2% at 1 year, and 83.8% at 2 years, with improved comorbidities. Radu Neagoe *et al*. reported %EWL of 41.8% at 3 months and 64.1% at 6 months, with favorable outcomes for type 2 diabetes mellitus (T2DM) and hypertension^13^. Complications include bleeding, leakage, abscess formation, and late complications like gastric stenosis, nutrient deficiencies, pouch migration, and exacerbated gastroesophageal reflux disease (GERD)^14^.

### Gastric bypass (Roux-en-Y)

Gastric Bypass (Roux-en-Y) is a multistep surgical procedure involving balloon sizing of the upper stomach, tract creation for lesser curvature dissection using hook cautery, and stomach division with an endoscopic stapler. It includes the use of a circular stapler anvil, enteroenterostomy with linear staplers, and retrocolic passage of the proximal Roux limb. A Penrose drain is used for this passage, and air leakage is checked during distension^15,16^. Roux-en-Y gastric bypass (RYGB) results in significant body weight loss due to reduced eating, increased energy expenditure, nutrient limitations, and potentially altered metabolic efficiency^17^. Weight loss averages around 77% of excess body weight at 1 year, with high-resolution rates for preoperative comorbidities, especially diabetes mellitus^17^. However, long-term weight loss varies between 34% and 80.2% %EWL 10 years post-surgery, and complications include wound infections, respiratory issues, hemorrhage, leakage, obstruction, hernia, gallstones, ulcers, and more^15,18,19^.

### Adjustable gastric banding (Lap-Band)

In the Adjustable Gastric Banding (Lap-Band) procedure, a Nathanson liver retractor is inserted through a 5-mm incision below the xiphoid to retract the left hepatic lobe. Two atraumatic graspers are used to manipulate the stomach and create a passage for the band around the stomach, with gastric-to-gastric sutures to align it above and below the band^20^. The adjustable gastric band induces weight loss by promoting early satiety and reducing appetite, impacting esophageal and proximal gastric functions through the vagus nerve^21^. Postoperative %EWL varies between 25.3 and 71.7%, with mean %EWL of 30% at 6 months, 40% at 12 months, 50% at 24 months, and 50–60% at 48 months in one study^21,22^. Long-term follow-up shows a mean BMI below 30 kg/m^2^ in patients followed for over 5 years, compared to a mean preoperative BMI of 42 kg/m^2^. Complications include gastric perforation, bleeding, port infection, conversion to open surgery, and death as early complications, and late complications such as band erosion, food intolerance, and access port problems^22^.

### Biliopancreatic diversion with duodenal switch (BPD/DS)

The biliopancreatic diversion procedure involves three main components: creating a stomach tube with pylorus preservation, performing distal ileoileal anastomosis, and establishing proximal duodenal-ileal anastomosis^11^. Initially, a SG is performed, followed by transection of the small bowel 250 cm proximal to the ileocecal valve. The alimentary limb is anastomosed to the transected portion of the duodenum to form the duodenoileal anastomosis, and an ileoileal anastomosis is created 100 cm proximal to the ileocecal valve. The procedure concludes with suturing the surgical wounds^11,23^. Biliopancreatic diversion results in early weight loss due to SG and long-term weight loss from fat malabsorption. Hormonal changes, including reduced ghrelin and increased peptide YY levels, lead to early satiety. Gastrectomy affects ghrelin changes, while rapid nutrient entry into the ileum and jejunum after the distal bypass increases peptide YY levels^11^. Despite being less common than other bariatric procedures, biliopancreatic diversion achieves impressive long-term weight loss, ~70% %EWL^24^. Common complications include anastomotic leak, hemorrhage, and nutritional deficiencies. Other complications involve abdominal wall hematoma, wound infections, atelectasis, cholecystitis requiring cholecystectomy, and trocar site hernia, which can be surgically repaired^25^.

## Comparative analysis of weight loss procedures

### Efficacy in achieving weight loss

The reviewed literature compared various weight loss procedures; among them, SG was a highly effective option for significant weight reduction (Table 1). Durmush *et al*.^12^ reported impressive average %EWL figures: ~67.1% at 6 months, 81.2% at 1 year, and 83.8% at 2 years post-surgery. Even in the long run, SG showed promise, with %EWL ranging from 41.8% among 179 patients to 64.1% among 14 patients observed at the 72-month follow-up^13^. Gastric bypass also demonstrated significant efficacy, achieving an average %EWL of 77% at 1 year and maintaining the results throughout the 60-month follow-up^16^. However, the average %EWL at 10 years after surgery showed a decreasing trend, ranging between 34 and 80.2%^18^. On the other hand, adjustable gastric banding yielded more modest outcomes compared to other procedures, resulting in a %EWL of 30% at 6 months and 50–60% at 48 months during the follow-up period^22^. Conversely, BPD/DS emerged as one of the most effective options, with an impressive average %EWL of ~70%, sustained even at long-term follow-ups^24^.

**Table 1:** Comparative analysis of weight loss procedures and their impact on patients.

Weight loss procedure	Efficacy in achieving weight loss	Impact on comorbidities	Patient selection and suitability	Factors influencing surgical decision-making
Sleeve gastrectomy (SG)	- %EWL: 67.1% (6 months), 81.2% (1 year), 83.8% (2 years)^12^	- T2D: resolution (71%), improvement (18%)	- Suitable for a wide range of individuals, including lower BMI and significant health risks^26^	- Medical history, BMI, surgical risks, patient preferences
	- %EWL range: 41.8–64.1% at 72 months follow-up^13^	- HTN: resolution (39%), improvement (33%)	- Less technically demanding, rapid recovery, lower adverse events^26^	- Lifestyle and commitment to long-term changes
Gastric bypass (RYGB)	- %EWL: 77% (1 year), 34% to 80.2% (10 years)^16,18^	- Clinical reversal of diabetes mellitus (98%)	- Recommended for higher BMI or inadequate weight loss with less invasive options^26^	- Desire for a reversible or permanent solution
	- Maintained results through 60-month follow-up^16^	- Resolution/improvement in other comorbidities	- Increased risks of dumping syndrome and marginal ulceration^26^	- Experience and expertise of the bariatric surgeon
Adjustable gastric banding	- %EWL: 30% (6 months), 50–60% (48 months)^22^	- Improvement in some comorbidities	- Suitable for patients preferring less permanent solution or with lower BMI^30^	- Assessment of patient’s suitability for a complex procedure
		- Outcomes may not be as significant as SG and RYGB	- Less invasive, reversible, and faster recovery^30^	- Vitamin replacements due to potential malabsorption
Biliopancreatic diversion with duodenal switch (BPD/DS)	- %EWL: ~70%^24^	- Resolution/improvement in comorbidities	- Highly effective for severe obesity and significant health risks^26^	- Surgical risks, patient preferences
	- Sustained efficacy at long-term follow-ups^24^	- Provides substantial health benefits to patients	- Complex procedure requiring specialized surgical skills^26^	- Patient commitment to dietary changes
Comparative studies				- Tailoring procedure to individual’s needs and health circumstances
	- BPD/DS shows greatest BMI change after 1 year, followed by RYGB and SG^26^			
	- BPD/DS demonstrates highest % total weight loss, followed by RYGB and SG^27^			
	- BPD/DS outperforms other procedures regarding weight loss outcomes^28^			
	- Systematic review shows no significant differences in midterm and long-term weight loss outcomes between LRYGB and LSG^29^			
	- Laparoscopic gastric bypass demonstrates superior weight loss compared to laparoscopic adjustable gastric banding^31^			
	- Laparoscopic gastric bypass shows greatest average BMI reduction compared to laparoscopic sleeve gastrectomy and laparoscopic adjustable gastric banding^32^			
Impact on comorbidities				
	- Sleeve gastrectomy: resolution of type 2 diabetes (71%), improvement (18%)^12^			
	- Sleeve gastrectomy: resolution of hypertension (39%), improvement (33%)^12^			
	- Gastric bypass: clinical reversal of diabetes mellitus in 98% of patients^16^			
	- BPD/DS: provides substantial health benefits to patients^26^			
	- RYGB shows highest resolution of GERD (1.88 times greater than SG)^26^			
	- BPD/DS shows greater probabilities of GERD resolution than SG (1.57 times greater)^26^			
	- RYGB and BPD/DS show equivalent probabilities of resolving OSAS (1.46 and 1.76 times higher than SG, respectively)^26^			
	- LRYGB has most significant outcomes for diabetes, hypertension, sleep apnea, and hyperlipidemia, followed by LSD and then LAGB^32^			

%EWL, percentage of excess weight loss; GERD, gastroesophageal reflux disease; HTN, hypertension; OSAS, obstructive sleep apnea; RYGB, Roux-en-Y gastric bypass; T2D, type 2 diabetes.

Comparative studies have also been conducted on various weight loss procedures. One study compared SG to RYGB and BPD/DS. BPD/DS showed the greatest BMI change after a year, followed by RYGB and SG^26^. Similar outcomes were observed in another study with BPD/DS demonstrating the highest % total weight loss, followed by RYGB and SG^27^. Further analyses revealed that BPD/DS outperformed other procedures regarding weight loss outcomes^28^. Contrarily, a systematic review showed no significant differences in midterm and long-term weight loss outcomes between Laparoscopic Roux-en-Y gastric bypass (LRYGB) and LSG^29^. Another study compared laparoscopic adjustable gastric banding (LAGB) and laparoscopic gastric bypass, showing better weight loss results favoring gastric bypass^30^. Other studies reported consistent findings, with laparoscopic gastric bypass demonstrating superior weight loss compared to LABG^31^. In a comprehensive study encompassing various hospitals, laparoscopic gastric bypass showed the greatest average BMI reduction compared to LSG and LABG^32^.

### Impact on comorbidities

BPD/DS and RYGB demonstrate the most favorable outcomes when examining the impact on obesity-related comorbidities. In the study by Durmush *et al*.^12^, SG contributed to the complete resolution and significant improvement of type 2 diabetes in 71 and 18% of patients, respectively. It also resulted in the resolution of hypertension in 39% and improvement in 33% of patients. Similar results were found in the study by Neagoe *et al*.^13^, with 65.8% experiencing resolution or significant improvement in type 2 diabetes. In contrast, gastric bypass showed clinical reversal of diabetes mellitus in an impressive 98% of patients^16^. Although adjustable gastric banding can improve comorbidities, the outcomes may not be as significant as SG and gastric bypass. BPD/DS stands out as a highly effective procedure, improving obesity-related comorbidities and providing substantial health benefits to patients.

Regarding GERD, RYGB demonstrated the highest resolution, according to Sudan *et al*., with probabilities 1.88 times greater than SG. Even after BPD/DS, the probabilities of GERD resolution were 1.57 times greater, possibly due to a larger pouch and acid and bile diversion further downstream. They also noted that BPD/DS and RYGB were best for resolving type 2 diabetes and hypertension, outperforming SG. When comparing the chances of resolving obstructive sleep apnea (OSAS), RYGB, and BPD/DS showed equivalent probabilities, 1.46 and 1.76 times higher, respectively, compared to SG^26^. In the Bariatric Surgery Network study comparing comorbidities, they reported resolution or improvement rates of 44%, 68%, and 79% after 1 year for LAGB, LSG, and LRYGB, respectively, in terms of hypertension. For diabetes, the rates were 44%, 55%, and 83% for LAGB, LSG, and LRYGB, respectively. In patients with hyperlipidemia, the rates were 33%, 35%, and 66% for LAGB, LSG, and LRYGB, respectively. For patients with OSAS, the rates were 38%, 62%, and 66% for LAGB, LSG, and LRYGB, respectively. For GERD, the rates were 64%, 50%, and 70% for LAGB, LSG, and LRYGB, respectively. They concluded that LRYGB had the most significant outcomes for diabetes, hypertension, sleep apnea, and hyperlipidemia, followed by LSG and then LAGB. However, for GERD, LSG appeared less effective compared to LAGB, and LRYGB^32^.

### Patient selection and suitability for different procedures

Bariatric surgery is generally not recommended for individuals with a BMI less than 35 kg/m^2^ and without obesity-related comorbidities (Table 2). However, each weight loss procedure is suitable for different patient profiles. SG is a versatile option suitable for a wide range of individuals, including those with a lower BMI and significant health risks who may not be candidates for more complex surgeries. SG is less technically demanding, with rapid recovery and lower incidences of adverse events, making it particularly useful in low-resource settings^26^.

**Table 2:** Patient selection and suitability for different weight loss procedures.

Weight loss procedure	Suitability and patient profiles	Surgical risks and considerations	Factors influencing decision-making
Sleeve gastrectomy (SG)	- Suitable for a wide range of individuals, including lower BMI and significant health risks^26^	- Less technically demanding, rapid recovery, lower adverse events^26^	- Medical history, obesity-related health conditions^25,26^
	- Particularly useful in low-resource settings^26^		- BMI^27^
Gastric bypass (RYGB)	- Recommended for higher BMI or inadequate weight loss with less invasive options^26^	- Increased risks of dumping syndrome and marginal ulceration^26^	- Surgical risks and patient preferences^28^
	- Patients on chronic NSAIDs for arthritis may require careful consideration^26^	- Severe perioperative complications, such as anastomotic leaks^30^	- Desire for a reversible or permanent solution
	- Daily multivitamin intake is necessary^26^	- Stomal stenosis, nutrient deficiencies, and internal hernia formation^30^	- Experience and expertise of the bariatric surgeon^30^
Adjustable gastric banding	- Suitable for patients preferring less permanent solution or with lower BMI^30^	- Less invasive, reversible, and faster recovery^30^	- Assessment of patient’s suitability for a complex procedure
	- May be more appropriate for individuals with fewer obesity-related health issues^30^	- Shorter hospital stays and less serious side effects^30^	
Biliopancreatic diversion with duodenal switch (BPD/DS)	- Highly effective for severe obesity and significant health risks^26^	- Requires specialized surgical skills^26^	- Surgical risks, patient preferences
	- Careful evaluation is necessary due to potential malabsorption^26^	- Highest incidence of adverse effects^26^	- Patient commitment to dietary changes^29^

NSAIDs, non-steroidal anti-inflammatory drugs; RYGB, Roux-en-Y gastric bypass.

Gastric bypass is recommended for patients with a higher BMI or those without adequate weight loss with less invasive options. Careful patient selection is crucial due to the increased risks of dumping syndrome and marginal ulceration associated with this procedure. Patients on chronic NSAIDs (non-steroidal anti-inflammatory drugs) for arthritis may need to be more suitable candidates. Additionally, daily multivitamin intake is necessary, so patient commitment is essential. The gastric bypass carries the risk of severe perioperative complications, such as anastomotic or staple line leaks, occurring in ~4% of cases and may require re-operation. Other potential adverse effects include stomal stenosis, nutrient deficiencies, and internal hernia formation, all of which must be considered during patient selection^26,30^.

Adjustable gastric banding may be more suitable for patients who prefer a less permanent solution or have concerns about more extensive surgeries. It is less invasive, reversible, and associated with less serious side effects, leading to faster postoperative recovery and shorter hospital stays. However, it may be more appropriate for individuals with a lower BMI and fewer obesity-related health issues^30^. BPD/DS is highly effective but typically recommended for patients with severe obesity and significant health risks. Careful evaluation is necessary to assess the patient’s suitability for this complex procedure, as it requires vitamin replacements due to potential malabsorption. Performing BPD/DS also demands specialized surgical skills, and it carries the highest incidence of adverse effects^26^.

### Factors influencing surgical decision-making

Several factors influence the choice of weight loss procedure for each patient. Medical history and obesity-related health conditions are significant determinants in selecting the most suitable option^25,26^. BMI is a crucial consideration, with higher BMI potentially indicating the need for more extensive procedures like gastric bypass or BPD/DS^27^. Surgical risks and patient preferences, including the desire for a reversible or permanent solution, also play a crucial role in the decision-making process^28^. Furthermore, the patient’s lifestyle and commitment to long-term changes, such as dietary modifications and regular exercise, are essential factors regardless of the procedure^29^. The experience and expertise of the bariatric surgeon are equally critical in selecting the most appropriate procedure for each patient, ensuring optimal outcomes and minimizing potential complications^30^. A comprehensive evaluation of these factors is essential to tailor the weight loss procedure to the individual’s needs and health circumstances.

## Emerging and investigational bariatric procedures

### Intragastric balloon

The Intragastric Balloon (IGB) is a minimally invasive weight management tool – a single silicone sphere filled with saline, endoscopically placed in the stomach^33^. It can remain for up to 6 months, requiring endoscopic removal, and is effective when combined with lifestyle modifications^34^. In a study of adults with BMIs from 30 to 40 kg/m^2^, one group received the IGB and lifestyle advice, while the other received lifestyle intervention alone. The IGB group had significantly greater short-term weight loss at 3 and 6 months after removal, but some experienced adverse gastrointestinal events, leading to early balloon removal. A small percentage showed gastric abnormalities at removal^35^. In a post-marketing clinical trial, 8.9% of adults with BMIs of 30–40 kg/m^2^ experienced device and procedure-related serious adverse events (SAEs) with the IGB, below the 15% threshold. Combining the IGB with lifestyle intervention led to significant weight loss, supporting its use as an adjunct for weight reduction, with lower SAE rates in real-world clinical settings^36^. IGB therapy for super-obese patients showed short-term weight loss of 9.04% and a BMI reduction of 3.8 at 60 months postoperatively. However, more durable weight loss was achieved when IGB was combined with definitive bariatric surgeries, though three deaths (1.4%) were reported in the study^37^.

### Heliosphere balloon

The Heliosphere balloon is an air-filled polyurethane IGB designed to reduce side effects like nausea and vomiting^38^. It is generally well-tolerated for the first 6 months but can pose technical challenges during insertion and removal. A study by Giuricin found that the Heliosphere BAG led to a mean weight loss of 12.66 kg and an overweight loss of 24.37% during a 6-month treatment period for severely obese patients. At 18 months post-removal, the mean BMI was 37.28 kg/m^2^, with a mean weight loss of 9.8 kg or 18.2%. The study suggested using the balloon as a ‘bridge treatment’ before major surgery to mitigate preoperative risks for severely obese patients^39^. Another study by Lecumberri *et al*. showed significant weight loss and BMI reduction with the Heliosphere IGB in 84 patients before bariatric surgery. Mean weight loss was 14.5 kg, BMI loss was 5.3 kg/m^2^, and 70.4% achieved a body weight loss of over 10%. The %EWL reached 33.2%. Age and initial BMI inversely affected weight loss outcomes^40^.

### Spatz gadget

The Spatz balloon is an adjustable IGB filled with saline, designed for adults with a BMI between 30.0 and 40.0 kg/m^2^ who have not succeeded in losing weight through supervised programs^41^. It is implanted endoscopically and can be adjusted during its 8-month usage period before endoscopic removal. In a clinical study, 92% of Spatz3 Adjustable Balloon recipients experienced at least a 5% reduction in total body weight, with an average weight loss of 15.0%^41^. However, it is not suitable for individuals with specific medical conditions. It should be part of a long-term supervised diet and behavior modification program to enhance weight loss chances after removal. A study by Russo *et al*. compared the Spatz Adjustable Balloon System (ABS) with the BioEnterics Intragastric Balloon (BIB) for temporary obesity treatment. Both procedures resulted in similar median weight loss and BMI reduction at the end of therapy. Complications during the procedure and removal were minimal, with some cases of intolerance, gastrectasia, vomiting, and bowel migration. Weight gains were observed in the Spatz ABS group compared to the BIB group during follow-ups^42^. In addition to the Spatz balloon, other IGB devices like the ReShape Duo dual balloon system, Obalon system, and Elipse balloon are effective for promoting weight loss^43–45^. These balloons reduce food intake, promote fullness, delay gastric emptying, and influence hunger and satiety hormones. The choice of balloon depends on individual characteristics and medical advice.

### AspireAssist device

The AspireAssist device is an option for adults with a BMI between 35.0 and 55.0 kg/m^2^ who have not succeeded in losing weight through nonsurgical means^46^. It involves a tube attached to a Skin-Port port outside the abdomen. After each of their three daily meals, patients open the port valve, connect tubing, and allow gravity to drain stomach contents into a toilet or container. In a 52-week trial, the AspireAssist group achieved a significant 12.1% body weight loss at 52 weeks compared to 3.5% in the Lifestyle Counseling group, with a difference of 8.6%. This procedure also led to better excess weight loss, improved cardiometabolic risk factors, and enhanced quality of life. It demonstrated a favorable safety profile and high procedural success. More research is needed for long-term effects and broader applicability^47^. Another study involved 201 participants who underwent aspiration therapy between 2012 and 2016, resulting in promising weight loss outcomes (mean total weight loss of 17.1 and 18.2% per protocol), improved cardiometabolic parameters, and minimal complications. However, some participants discontinued the therapy for various reasons, necessitating further research to understand its benefits and drawbacks^48^. In a shorter 4-week study, 25 obese individuals achieved a considerable weight loss of 16.5 ± 7.8 kg, suggesting that the AspireAssist device may be a valuable tool for managing obesity^49^.

### Endoscopic sleeve gastrectomy

Endoscopic Sleeve Gastrectomy (ESG) is a minimally invasive weight loss procedure that uses an endoscope to reduce the size and reshape the stomach into a tubular structure. This procedure can lead to calorie intake reduction and weight loss. It offers advantages like fewer complications, shorter hospital stays, and quicker recovery compared to traditional bariatric surgeries^50,51^. Abu Dayyeh *et al*. studied ESG’s efficacy and safety for treating classes 1 and 2 obesity. They found that ESG and lifestyle modifications led to significant and sustained weight loss up to 104 weeks and improved metabolic comorbidities. ESG is considered a safe and effective weight loss intervention for individuals with class 1 or 2 obesity, but more research is needed to assess its long-term durability and effectiveness in larger populations^52^.

Neto *et al*.’s study supported the short-term effectiveness and safety of ESG using the Overstitch system for patients with classes I and II obesity. They observed significant weight loss at 6 months and 12 months, along with substantial excess BMI loss. The safety profile was favorable, with minimal complications^53^. A systematic review comparing LSG to other bariatric procedures, such as LGB and LAGB, found that LSG was well-tolerated, had a lower complication rate than LGB, and showed promising short-term weight loss outcomes. LSG also demonstrated potential in treating T2DM^54^.

## Multidisciplinary approach to bariatric surgery

**Figure 1:**
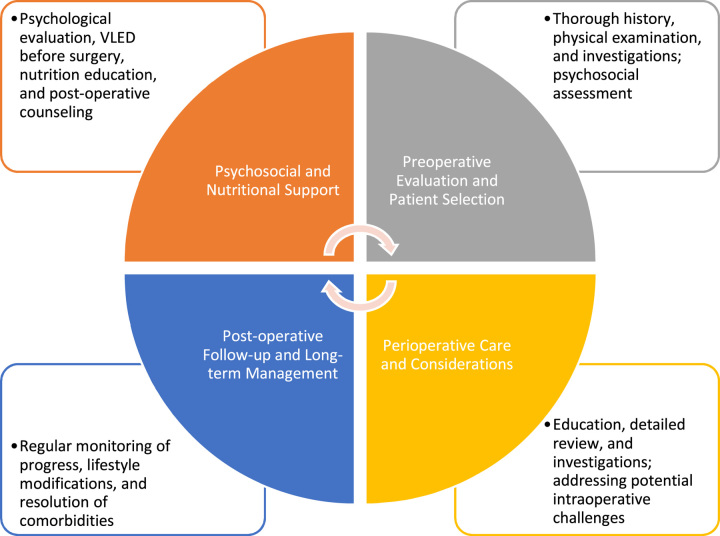
Multidisciplinary approach to bariatric surgery.

Bariatric surgery is a highly effective option for treating severe obesity, typically indicated for individuals with a BMI greater than 40 kg/m^2^ or 35 kg/m^2^ with obesity-related comorbidities^55^ (Fig. 1). This surgical intervention becomes crucial when nonsurgical methods fail to control the patient’s weight. However, the success of bariatric surgery goes beyond the surgical techniques employed; it necessitates a comprehensive and multidisciplinary approach to address the patient’s psychosocial and nutritional needs. A key aspect of this multidisciplinary approach involves a team of various healthcare professionals working collaboratively. This team typically comprises surgeons, nurses, clinical psychologists, dietitians, and other relevant specialists^56^. Their combined expertise is aimed at enhancing the surgical outcome and improving the patient’s overall quality of life^56^.

The multidisciplinary approach encompasses various stages of the patient’s journey through bariatric surgery. Thorough preoperative care is essential to assess the patient’s suitability for the procedure and to educate them about the surgery’s benefits and risks. Perioperative care involves meticulous attention to detail during the surgical procedure to ensure optimal outcomes and minimize complications. Postoperative care is equally crucial, involving close monitoring, pain management, and early detection of potential issues to promote a smooth recovery^56^. Furthermore, the multidisciplinary team provides vital nutritional and psychosocial support to the patient. This entails developing personalized dietary plans for weight loss and proper post-surgery nutrition. Psychosocial support is essential to address emotional and mental aspects related to the surgery, such as lifestyle changes, body image, and adjustment to new eating habits^56^.

### Preoperative evaluation and patient selection

Perioperative evaluation and patient selection are crucial in the multidisciplinary approach to bariatric surgery^56^. This involves a thorough history, physical examination, and assessment of comorbidities across various systems^57^. Dietitians play a vital role in monitoring nutritional status and facilitating some weight loss before surgery to enhance overall health^57^. Imaging studies and investigations, like abdominal ultrasound and esophagogastroduodenoscopy, are used to evaluate gastrointestinal comorbidities, while chest X-rays and electrocardiograms assess the cardiovascular system^57^. Additional assessments, such as stress echocardiography, may be conducted in specific cases to evaluate cardiovascular risks^58^.

Psychosocial assessment is another critical aspect, involving clinical interviews and objective tests to gauge mental and emotional well-being^59^. Although various assessment tools exist, preoperative psychosocial evaluation impacts long-term bariatric surgery outcomes, including adherence to lifestyle changes, emotional well-being, and weight loss^57^. The multidisciplinary team collaborates to select the most suitable bariatric procedure based on individual characteristics and goals^60^, ensuring a personalized approach for successful outcomes.

### Perioperative care and considerations

The multidisciplinary team ensures patient safety throughout the bariatric surgery journey^61^. They begin by educating and counseling the patient on surgical options, risks, outcomes, and the importance of postoperative lifestyle changes. Informed consent is obtained to confirm the patient’s understanding^61^. Before surgery, the team updates the patient’s history, conducts a comprehensive examination, and measures their current weight^61^. Various tests, such as pregnancy tests, blood glucose, and liver function tests, are performed, along with imaging and cardiac assessments, to detect and address any abnormalities^61^.

Certain situations can lead to the cancellation of bariatric surgery, including pregnancy, medical unfitness, unstable psychiatric conditions, ongoing substance addiction, and lack of informed consent^62^. Ensuring the patient’s suitability is essential for safety and positive outcomes. During surgery, the team is prepared for potential challenges, such as difficult intubation, proper patient positioning, managing hypoxia, and effective pain control^63^. The operating room bed is selected to support the patient’s weight, and additional safety measures are employed^63^.

Post-surgery, the patient progresses through dietary stages, transitioning from clear liquids to a regular diet over several weeks^63^. Close monitoring includes pain management, fluids, wound care, and deep venous thrombosis prophylaxis^63^. Encouraging early ambulation and initiating an exercise program are essential for the patient’s overall health and surgery success.

### Postoperative follow-up and long-term management

Postoperative follow-up is a critical aspect of the multidisciplinary approach to bariatric surgery^64^. After the surgery, the patient is placed on a personalized follow-up schedule established by the multidisciplinary team. This team consists of clinicians, clinical psychologists, and nutrition professionals, all of whom play essential roles in the patient’s postoperative care.

During the follow-up visits, clinicians conduct routine examinations to monitor the patient’s progress and assess the resolution of comorbidities. Clinical psychologists provide the necessary education and counseling to support the patient in adopting and maintaining lifestyle modifications following the surgery. Nutrition professionals work closely with patients to ensure they adhere to an optimum diet schedule that complements their weight loss journey^64^.

At each follow-up visit, the patient’s nutrition and weight loss progress is carefully evaluated, along with their adherence to lifestyle modifications and the resolution of any comorbidities. Regular assessments allow the multidisciplinary team to monitor the patient’s overall health and well-being and make any necessary adjustments to their treatment plan^64^.

Studies have shown that adherence to postoperative follow-up significantly impacts the clinical outcomes of bariatric surgery^64^. For instance, a study conducted by Spaniolas *et al*. evaluated the effect of postoperative follow-up on 12-month weight loss. They found that complete adherence to the follow-up program was independently associated with achieving excess (greater than or equal to 50%) or total (greater than or equal to 30%) weight loss^64^. This highlights the importance of regular follow-up in supporting the patient’s weight loss journey and achieving successful outcomes after bariatric surgery.

### Psychosocial and nutritional support

Psychological and nutritional support are essential for an effective bariatric multidisciplinary team^56^. Before bariatric surgery, candidates undergo a psychological evaluation to assess their readiness for the procedure and to address any potential psychosocial issues. Additionally, they are counseled to lose weight before surgery^56^. Registered dietitians play a crucial role in working with patients to develop personalized meal programs that meet their nutritional needs, facilitate weight loss, and promote overall health.

As obesity is considered a form of high-energy malnutrition, patients are often placed on a very low-energy diet (VLED) for 2–4 weeks before surgery. This approach helps reduce the size of the liver by 25%, thereby decreasing the risk of operative complications^65^. Educating patients about nutrition and its impact on successful weight loss is also vital to preoperative and postoperative care. Guiding food choices, portion control, and eating techniques empowers patients to make sustainable changes to their eating habits^65^.

Following the surgery, the multidisciplinary team continues to offer psychological support to ensure effective weight loss^66^. The postoperative follow-up serves as a platform to address psychosocial issues that may arise after bariatric surgery^67^. While the primary goal of bariatric surgery is to improve the quality of life for obese patients, not all patients who achieve weight loss experience significant improvements in their overall well-being^67^.

According to Kalarchian and Marcus^67^, some common psychosocial issues patients encounter after bariatric surgery include concerns about excess skin, social support changes, struggles with illicit drug use disorders, and severe depression and suicidal thoughts. Addressing these psychological challenges is crucial to supporting the patient’s long-term success and well-being after the surgery.

## Safety and complications in bariatric surgery

### Common postoperative complications

Bariatric surgery is a popular and effective option for obesity, modifying the digestive system to promote weight loss^5^. However, it is not without risks, with complications falling into early (immediate postoperative) and late (after 30 days) categories^68^. Early complications include infections, bleeding, anastomotic leaks, pulmonary embolism (PE), and gastrointestinal issues like obstructions or perforations^68^. Timely medical attention and potential surgical intervention are crucial to manage these. Late complications manifest beyond the immediate postoperative period and include nutritional deficiencies, gallstones, gastrointestinal ulcers, hernias, and internal herniation^68^. Nutritional deficiencies stem from reduced nutrient absorption in some procedures, requiring ongoing monitoring and supplementation.

While complications can occur, bariatric surgery is generally safe, with varying risks based on the procedure, patient health, and surgical team expertise. Preoperative assessments, patient selection, and postoperative care are vital for minimizing risks and optimizing outcomes. Patients considering bariatric surgery should undergo a thorough evaluation, receive counseling, and understand potential risks and benefits. Postoperative care and follow-up are essential for addressing complications and supporting the patient’s weight loss journey.

### Surgical site infection

Over the years, the field of surgery has made significant advancements in medical knowledge and infection control measures. However, surgical site infections (SSI) continue to be a persistent and concerning issue in healthcare institutions^69^. The prevalence of SSI is a matter of concern due to its association with increased nosocomial morbidity and mortality rates^69^. These infections can range in severity from relatively mild surface incisional infections to more serious complications, such as intra-abdominal abscesses involving deep organ spaces and the development of incisional hernias^70,71^.

The rise in SSI has been linked to the increasing prevalence of higher BMI in patients undergoing surgery, with a progressive increase in infection risk as BMI categories increase^72–74^. For instance, research by Kushner *et al*. found that patients with class I obesity have a 50% higher risk of SSI compared to individuals with a normal BMI. Patients classified as class II obese face an 86% higher risk, and patients with class III obesity experience a risk exceeding 100% higher than those with a normal BMI^72^. Several factors contribute to the risk of SSIs, including changes in respiratory physiology, modifications in the skin and soft tissue integrity, the presence of comorbidities like T2DM and cardiovascular disease, drug therapies, and antimicrobial resistance^68^.

Another study conducted by Christou *et al*.^71^ revealed that the incidence of wound infection following bariatric surgery varies, with reported rates ranging from as low as 1% in the International Bariatric Surgery Registry to as high as 16.5% in several published studies. The bacterial isolates found in the study encompassed a range of microorganisms, with *Staphylococcus aureus* accounting for 39% of the isolates, alpha-hemolytic *Streptococcus* at 26%, *Enterococcus* at 16%, *Proteus mirabilis* at 9%, and various other bacteria collectively comprising 10% of the isolates^71^.

Addressing the risk of SSIs in bariatric surgery and other procedures requires a comprehensive approach. Healthcare institutions and surgical teams must implement strict infection control measures, adhere to evidence-based guidelines, and tailor preventive strategies to address the unique risks associated with each patient. Proper preoperative evaluation, optimization of patient health, and meticulous postoperative care are essential to reducing the incidence of SSIs and improving overall surgical outcomes for patients.

### Urinary tract infections

Urinary tract infection (UTI) is a common postoperative complication of bariatric surgery, affecting over 40% of patients and accounting for a significant portion of nosocomial infections, with ~1.7 million patients affected annually^75,76^. UTIs can occur at any point along the urinary tract, including the urethra, bladder, ureters, or kidneys. During bariatric surgery, the use of urinary catheters is often necessary to facilitate urine drainage and monitor output. However, this practice also introduces potential risks, as catheters can serve as a pathway for nosocomial bacteria to enter the urinary tract, leading to UTIs^76^.

In ambulatory patients diagnosed with a UTI, *Escherichia coli* (*E. coli*) is the primary causative agent, responsible for over 90% of cases. Similarly, ~50% of nosocomial catheter-associated UTIs (CAUTIs) are attributed to *E. coli* infections^77^. The duration of urinary catheterization also plays a crucial role in UTI risk. Patients with catheters in place for more than 2 days are twice as likely to develop a UTI compared to those with catheterization lasting 2 days or less^76^. Apart from urinary catheter use, several other risk factors have been identified in previous studies. These factors include age, with older patients being more susceptible to UTIs, longer operative times during surgical procedures, and prolonged hospital stays, which increase exposure to healthcare-associated infections^75,76,78,79^.

To reduce the risk of UTIs following bariatric surgery, healthcare providers must adhere to strict infection prevention protocols. This may involve minimizing the duration of urinary catheterization whenever possible, using aseptic techniques during catheter insertion and maintenance, and employing prophylactic antibiotics when indicated. Additionally, healthcare professionals should be vigilant in monitoring patients for signs and symptoms of UTIs and promptly initiate appropriate treatment when infections are identified. By addressing these risk factors and implementing effective preventive measures, healthcare teams can help reduce the incidence of UTIs in bariatric surgery patients and improve overall postoperative outcomes.

### Gastrointestinal leaks

Gastrointestinal leaks, also known as anastomotic leaks or gastric leaks, are serious and potentially life-threatening complications that can occur after certain gastrointestinal surgeries, including bariatric surgeries. While the incidence of postoperative gastrointestinal leaks has decreased over the years, it remains a relatively infrequent complication that carries a significant risk of morbidity and mortality^80,81^.

The rates of leaks after gastrointestinal surgeries vary depending on the specific site of the anastomosis. For instance, leak rates are observed in the range of 2–16% for the esophagus, 1–9% for the stomach, 9–16% for the pancreas, 10–16% for the bile ducts, 1–3% for the small intestine, 3–29% for the colon, and 8–41% for the rectum^82^. It is important to note that the location of the anastomosis and the type of surgery performed can significantly impact the risk of leaks.

Gastrointestinal leaks can lead to various complications, including intra-abdominal abscesses, sepsis, and other infections, which can further contribute to morbidity and mortality. The severity of these leaks can range from mild and self-limiting to severe and life-threatening. Mortality rates associated with gastrointestinal leaks can be as high as 35%, emphasizing the importance of early detection and prompt intervention^82^.

To minimize the risk of gastrointestinal leaks, surgeons follow meticulous surgical techniques and employ advanced technologies, such as intraoperative leak testing, to ensure the integrity of the anastomosis. Additionally, postoperative monitoring and early recognition of signs and symptoms of leaks are crucial for timely intervention and management.

Patients undergoing gastrointestinal surgeries, including bariatric procedures, should be educated about the potential risks and signs of complications, including gastrointestinal leaks, so that they can seek medical attention promptly if needed. By adopting comprehensive preventive measures and maintaining a high index of suspicion for leaks in the postoperative period, healthcare teams can work together to reduce the incidence of gastrointestinal leaks and improve patient outcomes after bariatric and other gastrointestinal surgeries.

### Deep vein thrombosis

The reported incidence of venous thromboembolism (VTE), which includes PE or deep vein thrombosis (DVT), following bariatric surgery exhibits significant variability, ranging from 0% in some studies to as high as 3.5% in others^83,84^. A study carried out by Stein and Matta^83^ revealed that the in-hospital prevalences of PE, DVT, and VTE following bariatric surgery were found to be 0.9%, 1.3%, and 2.2%, respectively.

Obesity itself is a significant risk factor for VTE, particularly symptomatic DVT. The combination of obesity and abdominal surgery that necessitates general anesthesia for ~30 min or more further increases the risk of thrombosis, leading to a 10-fold increased risk for the development of VTE^85^. The mechanism behind this increased risk is attributed to factors such as venous stasis caused by abdominal adiposity, endothelial dysfunction, and the pro-inflammatory and prothrombotic state associated with obesity^85,87^.

Age is also a notable risk factor for adverse events following bariatric surgery, including VTE^86^. As patients undergoing bariatric procedures may include older individuals, advanced age can contribute to an increased risk of postoperative complications, including VTE.

To mitigate the risk of VTE following bariatric surgery, several preventive measures are implemented. These may include the use of pharmacological prophylaxis (such as anticoagulant medications) and mechanical prophylaxis (such as pneumatic compression devices) to enhance blood flow and prevent clot formation. Early ambulation and patient mobilization after surgery are also essential to reduce the risk of venous stasis.

It is crucial for the multidisciplinary team caring for bariatric surgery patients to identify those at higher risk for VTE and tailor the prophylactic measures accordingly. By implementing comprehensive VTE prevention strategies, healthcare providers can reduce the incidence of postoperative complications and optimize patient outcomes following bariatric surgery. Regular monitoring and prompt detection of any signs or symptoms of VTE can further contribute to early intervention and improved patient care.

### Long-term nutritional deficiencies and monitoring

Long-term nutritional deficiencies and monitoring are essential in managing patients who have undergone bariatric surgery. Weight loss procedures, such as gastric bypass, SG, and biliopancreatic diversion, can alter the gastrointestinal anatomy and the absorption of nutrients, causing deficiencies in iron, calcium, and various vitamins, including vitamins D, B1, B9, and B12^87^.

These deficiencies can lead to specific health conditions such as anemia due to iron deficiency, osteopenia (reduced bone density), and neurological symptoms resulting from vitamin B12 deficiency. Other malnutrition-related complications have been observed following gastric bypass surgery (GBP), such as protein malnutrition and vitamin A deficiency, which may cause ocular complications^87,88^.

Monitoring for postoperative deficiencies is crucial as they can arise anytime. However, most deficiencies are commonly observed between 12 and 15 months after the surgery, except for vitamin D3, which may occur earlier^89^. Among these deficiencies, iron, vitamin D3, and folic acid deficiencies were notably more frequent in the first year compared to the second year after the surgery^89^.

Various factors can contribute to postoperative nutritional deficiencies, including preoperative deficiencies, intolerance to certain foods after surgery, alterations in taste and eating patterns, and non-adherence to dietary and supplement recommendations post-surgery^90^. Early postoperative deficiencies often correspond with the most common preoperative deficiencies, suggesting that nutritional imbalances existing before the surgery may persist after the procedure. This highlights the importance of preoperative nutritional assessments and interventions to optimize the patient’s nutritional status before bariatric surgery^89^.

To address and prevent nutritional deficiencies, bariatric surgery patients should receive ongoing nutritional counseling and follow-up care from registered dietitians as part of the multidisciplinary team. The dietitians work closely with patients to develop personalized meal plans and supplement recommendations to ensure adequate intake of essential nutrients. Regular blood tests are typically conducted to monitor the patient’s nutritional status and detect deficiencies early, allowing prompt interventions.

Long-term nutritional monitoring and follow-up are crucial to ensuring bariatric surgery patients’ overall health and well-being and to minimize the risk of complications related to nutritional deficiencies. By actively addressing and managing nutritional issues, healthcare providers can enhance the success and safety of bariatric surgery as a weight loss and metabolic health intervention.

### Addressing adverse outcomes and patient safety

#### Preoperative evaluation/assessment

During the preoperative evaluation of patients considered for bariatric surgery, healthcare providers conduct a comprehensive assessment to determine their candidacy and identify potential factors that may impact the procedure’s success^91^. The evaluation typically includes psychological testing, nutrition evaluation, and medical assessment. Medical evaluation involves laboratory testing, including complete blood counts, metabolic profiles, coagulation profiles, ferritin levels, thyroid function testing, and lipid profiles^91^. For those undergoing malabsorptive procedures, vitamin B12 and fat-soluble vitamin levels may be considered^91,92^.

Pulmonary and cardiac assessments are also essential components of the preoperative evaluation. This may involve conducting chest radiographs, arterial blood gas measurements, pulmonary function tests, electrocardiograms, and stress tests to assess coronary artery disease. Given the prevalence of obesity-related sleep apnea, a sleep study may also be part of the evaluation^92^.

Preoperative testing for *Helicobacter pylori* infection is recommended for patients undergoing bariatric surgery. Positive *H. pylori* testing has been associated with a higher likelihood of abnormal endoscopy and postoperative marginal ulcers. If *H. pylori* infection is detected, preoperative therapy for eradication is advised^92^.

Furthermore, liver histology is assessed as obesity often presents with nonalcoholic fatty liver disease. Gastric bypass surgery has shown significant improvement in liver histology for most patients. Preoperative evaluation involves blood testing and imaging studies, such as ultrasonography, with a liver biopsy performed if cirrhosis is suspected^92,93^.

Psychosocial factors significantly impact the long-term outcomes of bariatric surgery, affecting adherence to postoperative lifestyle recommendations, emotional adjustment, and weight loss outcomes^91^. A thorough preoperative evaluation by bariatric behavioral clinicians is essential to identify risk factors affecting surgical success and weight loss goals. This evaluation fosters a trusting relationship between the clinician and the patient, allowing for tailored interventions and support throughout the weight loss journey^91,93^.

#### Postoperative outcome/care

Adherence to professional recommendations is a critical aspect of post-bariatric surgery care, and it encompasses following dietary guidelines, engaging in physical activity, attending medical follow-ups, and participating in support group sessions. Despite its importance, studies have shown that preoperative and postoperative noncompliance with these recommendations is relatively common^94^. However, adherence to these postsurgical recommendations has been closely linked to the procedure’s success in terms of weight loss^95^.

Nutritional care after bariatric surgery is paramount for successful wound healing and overall well-being^89^. During the first year after surgery, patients experience rapid weight loss, which puts them at risk for deficiencies in vitamins and minerals, dehydration, and gastrointestinal symptoms^87,88^. Proper dietary management and supplementation are essential to prevent complications arising from these deficiencies and to ensure overall patient safety and well-being^88^. Regular screenings for anemia, clotting abnormalities, and nutrient deficiencies are also vital to detect any potential imbalances or deficiencies that could negatively impact the patient’s health^95^.

Monitoring weight loss progress is an essential part of post-bariatric surgery care. Unsuccessful weight loss or significant weight gain may signal potential issues that require additional support and prompt intervention from the healthcare team^95^. Addressing these concerns early can improve outcomes and ensure patients achieve their weight loss goals.

Engagement in physical activity is another crucial aspect of post-bariatric surgery care. Regular exercise can enhance weight loss, improve cardiovascular health, and improve the patient’s overall well-being. Healthcare providers should encourage patients to adopt an active lifestyle and provide guidance on suitable exercise routines based on their needs and capabilities.

Participation in support group sessions can also benefit patients undergoing bariatric surgery. Support groups offer a valuable platform for patients to share their experiences, challenges, and successes with others who have undergone similar procedures. These groups can provide emotional support, practical advice, and a sense of community, positively impacting the patient’s overall experience and long-term success.

## Patient outcomes and quality of life

### Weight loss and maintenance

The study conducted by Ryder *et al*. demonstrated that many participants who underwent bariatric surgery could maintain their weight loss long-term, with almost half of the individuals maintaining their weight within 20% of the lowest weight achieved after the surgery. This long-term weight maintenance was superior to the outcomes observed in a nonsurgical comparison group of individuals with severe obesity^96^. This highlights the effectiveness of bariatric surgery as a long-term weight control strategy.

Regular and continuous communication between patients and healthcare providers is essential to ensure successful long-term weight maintenance.^97^ In a study by Svetkey *et al*., participants who received personalized contact through monthly telephone or face-to-face interactions with healthcare professionals demonstrated significantly better weight loss retention compared to those in self-directed or interactive technology intervention groups^98^. This emphasizes the importance of ongoing support and guidance from healthcare professionals in helping patients sustain their weight loss efforts.

Consistent engagement in physical activity is also recognized as a key strategy for ensuring long-term weight maintenance. The study by Evans *et al*.^99^, which focused on patients who underwent RYGB, revealed that those who engaged in at least 150 min per week of moderate-intensity physical activity experienced greater weight loss at 6 and 12 months after the surgery. Physical activity contributes to weight loss, offers numerous health benefits, and supports overall well-being.

### Resolution of comorbidities

Bariatric surgery has been shown to promote significant weight loss and lead to substantial improvements or even resolution of obesity-related comorbidities. Conditions such as T2DM, hypertension, and cardiovascular diseases, which are often associated with obesity, have been found to improve after bariatric surgery.^100^

A retrospective cohort study conducted by Arterburn *et al*. in 2012 investigated the impact of gastric bypass surgery on adults with uncontrolled or medication-controlled type 2 diabetes. Over 13 years, 4434 adults underwent gastric bypass, and within 5 years after surgery, ~68.2% of them experienced complete remission of diabetes^101^. This study demonstrated the significant potential of bariatric surgery in achieving diabetes remission and improving glycemic control.

Hypertension is another common comorbidity associated with obesity, and weight loss-induced resolution of hypertension can significantly reduce cardiovascular risk in patients. Vogel *et al*.^102^ conducted a study that showed that achieving the resolution of hypertension through weight loss reduced the risks of coronary arterial disease (CAD) by ~39% in men and 25% in women. This highlights the cardiovascular benefits of weight loss achieved through bariatric surgery.

### Impact on mental health and well-being

Bariatric surgery has been associated with decreased prevalence and improvement in the severity of various mental health conditions, focusing on improvements in depression.

A study conducted by Dawes *et al*.^103^ found that among individuals seeking and undergoing bariatric surgery, the most prevalent mental health conditions were depression, with an estimated prevalence of 19%, and binge eating disorder, with an estimated prevalence of 17%. However, the study also revealed that bariatric surgery consistently correlated with postoperative reductions in the prevalence of depression, with improvements ranging from 8% to 74%. Notably, the severity of depressive symptoms also showed improvement following the surgical procedure^103^.

The observed improvements in depression and other mental health conditions after bariatric surgery can significantly impact patients’ overall well-being and quality of life. Weight loss, changes in hormonal factors after surgery, and increased self-esteem and body image perception have been suggested as potential factors contributing to improving mental health outcomes^103^.

### Limitations and future directions

In the context of this review on recent advancements in bariatric surgery, it is crucial to acknowledge certain limitations arising. Bariatric surgery research presents a diverse landscape encompassing various study designs, patient groups, and chosen outcome measures^104^. While this diversity aptly mirrors the intricate nature of the field, it simultaneously introduces complexities when attempting direct comparisons and generalizing findings across disparate studies^104^. Therefore, a promising direction for future research involves adopting standardized protocols and rigorous, expansive trials to enhance the reliability and quality of the presented evidence.

The immediate benefits observed in several modern bariatric procedures are undeniably compelling^105^. However, the critical factor of long-term sustainability necessitates deeper exploration. Attaining a comprehensive understanding of the durability of weight loss, metabolic enhancements, and potential complications over extended periods is crucial to guide informed clinical decision-making. Beyond conventional clinical endpoints, the incorporation of patient-centered outcomes assumes heightened significance. While achieving weight loss and metabolic improvements remains pivotal, an equally vital facet involves comprehending the broader impact of these procedures on aspects such as overall quality of life, psychological well-being, and patient satisfaction. This holistic perspective promises a deeper understanding of the intricate implications of bariatric surgery on individuals.

Future research endeavors would benefit from robust comparative studies to inform tailored and personalized surgical decisions. Directly comparing different techniques can offer a nuanced understanding of distinct advantages and disadvantages, aiding practitioners in making informed choices based on patient profiles. Similarly, recognizing that the effectiveness of bariatric surgery extends beyond the operating room, the integration of multidisciplinary care gains prominence. Collaborative efforts involving nutritionists, psychologists, and exercise specialists stand poised to optimize patient outcomes. Therefore, further research investigating the seamless integration of these components into the overarching bariatric care framework is warranted.

Technological advancement holds considerable promise for refining bariatric procedures, encompassing innovative surgical tools, advanced imaging methods, and minimally invasive approaches^106^. The potential of technology to reshape procedural success rates and patient recovery experiences beckons further exploration, making the study of these technological innovations and their tangible impact a compelling avenue for future research. In addition, the persistent challenge of health disparities demands continuous attention in bariatric surgery^107^. To this end, dedicated research aimed at dissecting and mitigating factors contributing to differential access, treatment outcomes, and overall experiences among diverse patient populations remains imperative.

## Conclusion

Bariatric surgery encompasses a range of well-established weight loss procedures, each with distinct mechanisms and outcomes. Additionally, emerging options like IGBs, AspireAssist devices, and endoscopic sleeve gastroplasty show promise but require further research to solidify their effectiveness. Nevertheless, bariatric surgery has consistently demonstrated substantial benefits in weight loss, comorbidities resolution, and quality of life improvements. By tailoring procedures to individual needs and continuing research efforts, the field is poised for advancement, leading to improved patient outcomes and transformative changes in the lives of those seeking effective weight loss solutions.

## Ethical approval

Ethics approval was not required for this review.

## Consent

Informed consent was not required for this review.

## Sources of funding

None.

## Author contribution

N.A.: conceptualization. All authors were involved in writing the manuscript.

## Conflicts of interest disclosure

All authors declare no conflicts of interest.

## Guarantor

Nicholas Aderinto.

## Data availability statement

Data sharing is not applicable to this article.

## Provenance and peer review

Not commissioned, externally peer-reviewed.
